# Pirfenidone restores metabolic hormones and cardiac autophagy via p-AMPK in MASH

**DOI:** 10.1186/s12967-026-08828-1

**Published:** 2026-08-19

**Authors:** Daniel López-Cifuentes, Ana Sandoval-Rodríguez, Rebeca Escutia-Gutiérrez, Juan Armendariz-Borunda, Jorge Gutiérrez-Cuevas

**Affiliations:** 1 Institute for Molecular Biology in Medicine and Gene Therapy, Department of Molecular Biology and Genomics, University of Guadalajara, CUCS, Guadalajara, Jalisco México; 2 Doctorate in Sciences in Molecular Biology in Medicine, University Center of Health Sciences, University of Guadalajara, Guadalajara, 44340 Jalisco Mexico; 3 Tecnologico de Monterrey, Campus Guadalajara, Jalisco, México

**Keywords:** Obesity, Glucolipotoxicity, MASH, C57BL/6J mice, H9c2 cells, Pirfenidone, Metabolic hormones, Autophagy, p-AMPK

## Abstract

**Background:**

Obesity promotes chronic inflammation and insulin resistance, disrupting key metabolic hormones (e.g., insulin and leptin) and triggering metabolic dysfunction-associated steatohepatitis (MASH). Obesity is commonly associated with cardiovascular disease. Autophagy preserves heart function in response to various stresses. Pirfenidone (PFD) is an antifibrotic and anti-inflammatory drug. However, its effects on the regulation of metabolic hormones and cardiac autophagy in MASH have not been investigated.

**Methods:**

Male C57BL/6J mice were fed a high-fat/high-carbohydrate (HFHC) diet for 16 weeks to induce obesity and MASH. From week 8, a subgroup received PFD (300 mg/kg/day) via gavage. In vitro, H9c2 cardiomyocytes were exposed to glucolipotoxicity to simulate metabolic stress. Serum metabolic hormones, cardiac histology, microarray analysis, and autophagy-related mRNA and protein levels were analyzed.

**Results:**

PFD treatment restored metabolic hormone balance and promoted cardiac autophagy in obese MASH mice. Mechanistically, PFD upregulated p-AMPK protein levels (*P* < 0.05), reversing the dysregulation of autophagy-related proteins both in vivo and in glucolipotoxicity-exposed H9c2 cells. Concurrently, PFD prevented systemic insulin resistance (*P* < 0.001), hepatic steatosis (*P* < 0.001), and liver damage (ALT/AST, *P* < 0.05). In cardiac tissue, PFD attenuated metabolic stress-induced hypertrophy (*Nppa/Nppb* mRNA), inflammation (*Tnf*,* Il6*,* Adgre1*, and *Ccl2* mRNA, *P* < 0.05), and fibrosis (*Tgfb1*,* Col1a1*, and *Col3a1* mRNA, *P* < 0.05).

**Conclusions:**

Pirfenidone has cardioprotective effects in obesity-associated MASH by restoring metabolic hormone levels and activating autophagy via p-AMPK signaling. These findings suggest a potential translational therapeutic strategy for treating cardiovascular complications in MASH.

**Supplementary Information:**

The online version contains supplementary material available at 10.1186/s12967-026-08828-1.

## Introduction

Obesity is characterized by the excessive accumulation of body fat and is a growing public health concern due to its association with insulin resistance, type 2 diabetes (T2D), metabolic dysfunction-associated steatohepatitis (MASH), and cardiovascular disease (CVD) [1, 2]. An excess of free fatty acids and triglycerides in obese individuals leads to ectopic lipid accumulation in non-adipose organs such as the liver, heart, and skeletal muscle. This ectopic deposition triggers lipotoxicity and promotes cellular stress, inflammation, and insulin resistance. The persisting chronic inflammation and oxidative stress in the visceral adipose tissue reduce the production of adiponectin and increase the secretion of resistin, leptin, plasminogen activator inhibitor-1 (PAI-1), and other pro-inflammatory adipokines and cytokines, thus exacerbating insulin resistance and contributing to atherosclerosis, cardiovascular stiffness, and diastolic dysfunction [2, 3]. Glucose-dependent insulinotropic polypeptide (GIP) (secreted by the gut) stimulates insulin secretion after meals and facilitates fat storage in adipose tissue [4]. Hyperglucagonemia is a hallmark of obesity and insulin resistance, which stimulates hepatic glucose production and serves as a critical factor in the development of T2D [5]. The high-fat/high-carbohydrate (HFHC) diet (also referred to as the Western diet) induces obesity, T2D, MASH, and CVD [6–8]. In C57BL/6J mice, the Western diet increases body weight, fasting glucose levels, and dyslipidemia [6, 7, 9]. Accumulation of lipids increases heart mass and alters cardiac morphology and function because of glucose intolerance, low-grade inflammation, and impaired insulin sensitivity, causing cardiomyocyte hypertrophy and interstitial fibrosis [7, 10–12].

Autophagy is classified into three primary types: macroautophagy, microautophagy, and chaperone-mediated autophagy. Macroautophagy (hereafter referred to as autophagy) promotes the degradation of defective organelles, excess proteins, and lipid droplets, thus maintaining homeostasis in multiple organs, including the heart [13, 14]. Autophagy is reduced in obesity and diabetes, thus contributing to the pathogenesis of cardiovascular disease. In the heart, the inhibition of autophagy causes premature cardiac aging, which drives hypertrophy, fibrosis, and impaired mitochondrial function, leading to progressive cardiac dysfunction [13, 15]. Inhibition of autophagy promotes pro-inflammatory gene expression, including *Il1b*,* Il6*, and interleukin (IL)-8 in the adipose tissue of obese mice, suggesting that autophagy may serve to dampen inflammation and thus limit the excessive inflammatory response during obesity [13, 14]. AMP-activated protein kinase (AMPK) is involved in the activation of autophagy when there is a decrease in nutrients, and activated AMPK levels are downregulated in a high-fat diet (HFD)-induced cardiac response [13–15]. Concurrently, key post-translational modifications and nutrient-sensing enzymes, such as sirtuins and protein acetylation, closely interact with AMPK pathways to govern metabolic flexibility and cellular survival under lipotoxic stress [16].

Pirfenidone (PFD) has anti-inflammatory, antioxidant, and anti-fibrotic properties in the lungs, liver, and heart [17–19]. We previously demonstrated that prolonged-release pirfenidone (PR-PFD) acts as an agonistic ligand for PPARα, decreasing hepatic steatosis and fibrosis, and preventing obesity-induced cardiac steatosis and fibrosis in a mouse MASH model [7, 20]. Crucially, while those findings highlighted the cardiovascular benefits of the prolonged-release formulation, the specific impact of standard PFD on systemic metabolic hormones and cardiac autophagy restoration had not previously been examined. To address this critical knowledge gap, we hypothesized that male C57BL/6J mice fed a Western diet would develop obesity-associated MASH, leading to a dysregulation of metabolic hormone profiles and the inhibition of cardiac autophagy, both of which could be reversed by standard PFD. Therefore, the aim of this study was to investigate the effects of standard PFD on systemic metabolic hormone homeostasis and cardiac autophagy restoration using in vivo (male C57BL/6J mice) and in vitro (glucolipotoxicity-exposed H9c2 cells) MASH models.

## Methods

### Animal care

The Institutional CUCS Research Committee at the University of Guadalajara approved this study (No. CI-01419). Animal care and experimental procedures were conducted in accordance with the ARRIVE guidelines [7].

### Experimental design in C57BL/6J mice

At 20 weeks of age, mice were randomly assigned to two groups: a normal diet group (ND, *n* = 7) and a high-fat/high-carbohydrate diet group (HFHC, *n* = 14). ND was composed of 18% kcal from fat (3.35 kcal/g), while the HFHC diet consisted of 45% kcal from fat (5.35 kcal/g) supplemented with an aqueous carbohydrate solution (42 g/L; composed of 55% fructose and 45% sucrose) to simulate a Western diet. At the end of the eighth week of feeding, seven HFHC mice were administered standard PFD (300 mg/kg/day) via gavage, dissolved in a 0.5% methylcellulose vehicle solution (Tecoland Corporation) (HFHC + PFD, *n* = 7). The selected dose was based on a previous study [20]. Food and water consumption were monitored three times a week by weighing the remaining amounts. Total caloric and energy intakes were calculated based on 3.35 kcal/g for the ND mice and 5.35 kcal/g for the HFHC mice, with the caloric contribution of the carbohydrate solution included in the final calculations.

### Insulin tolerance test

The insulin tolerance test (ITT) was performed as previously described [7]. Briefly, mice were fasted for 4 h, and basal blood glucose was determined from the tail vein. Each mouse received an intraperitoneal injection of human-recombinant short-acting insulin (0.025 IU/mouse) in 100 µL of saline. Blood glucose levels were measured at 30, 60, and 90 min post-injection using a glucometer, and the total area under the curve (ITT-AUC) was calculated.

### Organs and tibia dissection

Mice were anesthetized using tiletamine/zolazepam (15 mg/kg/body weight, Zoletil^®^ 50, Virbac), and the hearts, livers, and epididymal adipose tissue were isolated. Prior to excision, each heart was perfused through the left ventricle with 5 mL of ice-cold 1X PBS. The atria were removed, and the ventricles were sliced into three sections; one was fixed in 4% paraformaldehyde (0.1 M PBS, pH 7.4) and embedded in paraffin. The other two ventricular portions were snap-frozen on dry ice and stored at -80 °C to extract total protein and total RNA, respectively. Liver and epididymal fat tissues were also embedded in paraffin. The left tibia-fibula complex was isolated for biometric measurement.

### Serum biomarkers and histological analysis

Serum hormones were determined using the Bio-Plex Pro™ Mouse Diabetes 8-Plex immunoassay (#171F7001M, Bio-Rad Laboratories). Circulating ALT, AST, total cholesterol, triglycerides, low-density lipoprotein cholesterol (LDL-C), and very low-density lipoprotein cholesterol (VLDL-C) levels were measured using a Vitros-250 dry chemistry analyzer (Ortho-Clinical Diagnostics). Serial sections from ventricular, liver, and epididymal adipose tissues were cut at 4–5 μm and processed according to standard hematoxylin-eosin (H&E), Masson’s trichrome, and Picrosirius red staining (Sigma-Aldrich, Merck). Digital images (Olympus BX51 microscope) of the tissues were assessed in random fields (x20 magnification) per mouse using Image-Pro Plus 6.0 software.

### RNA isolation and RT-qPCR assays

The total RNA from ventricular tissue and H9c2 cells was isolated using TRIzol reagent (Invitrogen). RNA integrity was verified on a 1% agarose gel, and the concentration was quantified using a NanoDrop spectrophotometer (Thermo Fisher Scientific) [7]. A list of the specific primer sequences used is provided in Supplemental Table S1. The quantitative PCR reactions were run on a QuantStudio 5 Real-Time PCR System (Applied Biosystems). *Rn18s* and *Gapdh* were used as reference housekeeping genes.

### Western blotting

Snap-frozen ventricular tissue and H9c2 cells were homogenized in RIPA buffer; 50 µg of total protein was separated via SDS-PAGE gels and transferred to polyvinylidene difluoride (PVDF) membranes (Bio-Rad Laboratories). See reference for details [7]. A list of used antibodies is provided in Supplemental Table S2. GAPDH was used as the internal loading control.

### H9c2 cardiomyoblast culture and reagents

H9c2 cells were purchased from the American Type Culture Collection (ATCC, CRL-1446) and cultured in high-glucose Dulbecco’s Modified Eagle’s Medium (DMEM) supplemented with 10% fetal bovine serum (FBS, Thermo Fisher Scientific), and incubated in a humidified atmosphere with 5% CO_2_ at 37 °C. The medium was changed every 2–3 days, and cells were split using 1X TrypLE™ Select Enzyme (Thermo Fisher Scientific) at a ratio of 1:3 upon reaching confluence. All experiments were carried out at passages 7–15. To ensure a consistent response to metabolic stress, p-AMPK signaling, and autophagy regulation, cells were maintained and utilized strictly in their undifferentiated cardiomyoblast state.

The reagents used were dissolved as follows: PFD in 1X PBS at 10 mM, D-(+)-glucose (Glc) in DMEM at 1 M, dorsomorphin or compound C (CC) in dimethyl sulfoxide (DMSO) at 10 mM, and bafilomycin A1 (BafA1) in DMSO at 10 µM (all from Sigma-Aldrich, Merck). Palmitic acid (PA, Sigma-Aldrich, Merck) was solubilized in 0.1 N sodium hydroxide and combined with 5% bovine serum albumin (BSA) at a 6:1 volume ratio) to achieve a final stock concentration of 5 mM. All compounds were stored at -20 °C. The experimental concentrations and treatment durations for BafA1 and CC in vitro were selected based on well-established literature values and further optimized using preliminary MTT cell viability assays to ensure effective pathway modulation without inducing cytotoxicity.

### H9c2 cell treatments

H9c2 cells (70–80% confluence) were seeded at a density of 7.5 × 10^3^ cells/well in a 96-well plates for cell viability analyses, and at 5 × 10^5^ cells/well in 6-well plates in duplicate for RT-qPCR and western blot assays. Cells were initially seeded in 10% FBS-DMEM for 24 h; subsequently, the medium was changed to 1% FBS-DMEM medium overnight for serum starvation, followed by treatment with the respective compounds in serum-free DMEM. Briefly, PA was heated at 60 °C for 30 min and other compounds at 37 °C for 5 min; cells were pretreated with PFD and/or CC for 30 min and BafA1 for 1 h before exposure to PA and/or Glc for 24 h.

### Cell viability assay

Cell viability was assessed using the MTT [3-(4,5-dimethylthiazol-2-yl)-2,5-diphenyltetrazolium bromide] assay (Sigma-Aldrich, Merck). After the respective treatments, the cell culture medium was supplemented with 25 µL of MTT solution (5 mg MTT/mL in 1X PBS) at 37 °C for 3 h incubation. Thereafter, the medium containing MTT was removed, and the formed formazan crystals were dissolved in 100 µL of DMSO followed by incubation at 37 °C for 15 min. The absorbance was measured at 570 nm using a Quant microplate spectrophotometer (Bio-Tek Instruments Inc.). All measurements were performed in triplicate, and the data are presented as percentages relative to the control group.

### Oil red O staining

H9c2 cells were seeded at a density of 4 × 10^4^ cells/well in 24-well plates. After treatment with PA and/or PFD, the cells were fixed in 10% formalin (300 µL) for 10 min. Subsequently, cells were washed three times with PBS and stained with an Oil Red O (ORO; Sigma-Aldrich, Merck) working solution for 30 min at room temperature. The ORO stock solution was prepared by dissolving 150 mg of ORO powder in 50 mL of 60% isopropanol, whereas the ORO working solution was prepared by diluting the ORO stock in sterile distilled H_2_O (3:2 volume ratio). Cells were washed four times with H_2_O, counterstained with hematoxylin for 60 s, and rinsed thoroughly with H_2_O. Stained cells were photographed using an Olympus BX51 microscope.

For the quantification of lipid accumulation in H9c2 cells, digital images from four non-overlapping, technically homogeneous fields per experimental condition were captured at 20× magnification. The quantitative analysis was performed by measuring the percentage of Oil Red O-positive stained area relative to the total field area using Image-Pro Plus 6.0 software. To rule out any potential bias derived from variations in cell density across treatment groups, the thresholded lipid-positive area was strictly normalized relative to the total cell number per field, rather than the absolute background area.

### Transcriptome analysis

Total RNA was labeled for hybridization according to standard dual-channel microarray protocols. Heart transcriptome profiling was performed using dual-channel microarrays covering the complete Mus musculus genome (approximately 22,000 genes). To minimize biological variation and ensure sufficient transcript concentration, heart transcriptome profiling was conducted using pooled RNA samples, with each experimental group represented by a pool of equal amounts of total RNA extracted from three independent mice (*n* = 3 pooled biological samples per group). Microarray scanning and raw data acquisition were performed by the Microarray Unit of the Universidad Nacional Autónoma de México. Image processing and normalization were performed using *genArise* software. Differentially expressed genes (DEGs) were identified based on global Z-score thresholds (considering values > 1.5 as upregulated and < − 1.5 as downregulated). The selection of a Z-score cutoff was preferred over False Discovery Rate (FDR) corrections due to the targeted exploratory nature of this specific dataset and to avoid over-filtering of physiologically relevant fold-changes in baseline metabolic stress pathways, as optimized by the platform’s standardization guidelines. To determine shared and unique DEGs between experimental groups, Venn diagrams were constructed separately for the upregulated and downregulated gene sets. Gene lists were subsequently curated and organized using the *dplyr*,* tidyr*, and *readxl* R packages. Venn diagrams were then generated using the *VennDiagram* package, and graphical elements were arranged using the *grid* package.

Kernel density plots representing the global distribution of Z-scores were constructed using the *ggplot2*. Data wrangling and merging procedures were performed using *dplyr* and *tidyr*; additional layout tools such as *grid* or *gridExtra* were utilized only when external graphical objects (grobs) or legends required customized assembly.

Furthermore, process-organized gene maps and heatmaps were generated to display transcriptional profiles stratified by specific biological processes. Data frames were prepared using *dplyr* for heatmap construction, and visualization was performed primarily with *ggplot2*, incorporating the *grid* package for fine adjustments in panel spacing (e.g., via the *unit* function). Finally, functional enrichment analysis of DEGs was conducted using DAVID Bioinformatics Resources with annotations based on the Gene Ontology (Biological Process category). The raw and normalized microarray data have been deposited in the NCBI Gene Expression Omnibus (GEO) database and are publicly available under the GEO accession number GSE337556.

### Statistical analyses

Data were evaluated for normal distribution using the Shapiro-Wilk and Kolmogorov-Smirnov tests, while homogeneity of variance was strictly verified using Brown-Forsythe and Bartlett’s tests. Statistical significance was determined for parametric data using the Student’s *t*-test for two-group comparisons (with Welch’s correction applied only in cases of unequal variances), or one-way analysis of variance (ANOVA) followed by Tukey’s *post hoc* multiple comparison test for multi-group analyses. The non-parametric Mann-Whitney *U* test or Kruskal-Wallis test followed by Dunn’s *post hoc* test was applied to datasets that were not normally distributed. All quantitative data are expressed as the mean ± standard error of the mean (SEM). Statistical analyses were performed using GraphPad Prism software (version 9.0; GraphPad Software Inc.). A *P* value < 0.05 was considered statistically significant.

## Results

### PFD prevents the alteration of the anthropometric parameters, glucose levels, serum lipids, and biomarkers in obese mice

**Fig. 1:**
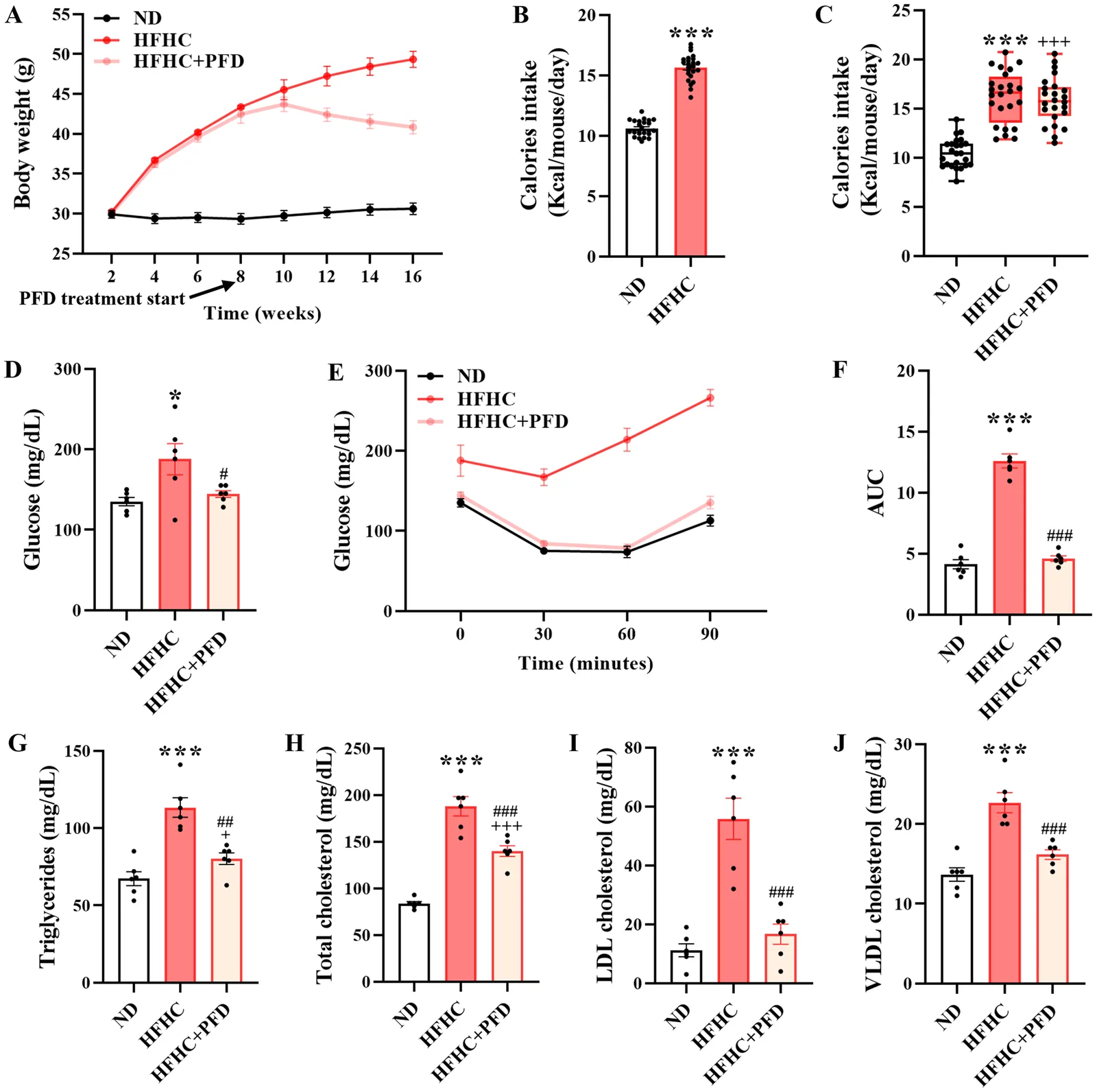
Effects of PFD on body weight, caloric intake, glucose levels, and serum lipid profile. **A**) Body weight during the experimental period (*n* = 7 mice per group); the arrow black marks the initiation of standard PFD treatment at week 8. Evaluation of definitive weight changes and statistical significance is presented in Table 1. **B**-**C**) Daily calorie intake between groups (Student’s t-test with Welch’s correction: *n* = 24 per group, 1–8 weeks; and one-way ANOVA followed by Tukey’s post hoc test: *n* = 24 per group, 9–16 weeks). **D**) Fasting blood glucose levels. **E**) Glucose tolerance test (GTT) curves. **F**) Area under the curve (AUC). **G**) Serum triglycerides. **H**) Total cholesterol. **I**) LDL cholesterol. **J**) VLDL cholesterol levels. Data are expressed as mean ± standard error of the mean (SEM). **D**-**J**) One-way ANOVA followed by Tukey’s post hoc test, *n* = 6 per group. *P < 0.05, ***P < 0.001 vs. ND; ^#^*P* < 0.05, ^###^*P* < 0.001 vs. HFHC; ^+++^*P* < 0.001 vs. ND

Mice fed an HFHC diet developed obesity and increased visceral fat, but mice treated with PFD at week 8 showed reductions in body fat mass, including decreased hepatic steatosis and heart weight (Supplementary Fig. S1). Furthermore, PFD-treated mice exhibited no adverse effects. All HFHC mice showed a steady increase in body weight over 16 weeks; however, as shown in Fig. 1a, the initiation of PFD intervention significantly restricted body weight gain during the later weeks of the protocol.

**Table 1:** Effects of the HFHC diet and PFD on parameters

Anthropometric measurements			
**Parameter**	**ND** (***n* = 7)**	**HFHC** (***n* = 7)**	**HFHC + PFD** (***n* = 7)**
Final body weight	30.85 ± 1.07	49.61 ± 1.35^***^	40.57 ± 1.35^###^
Liver weight (g)	1.51 ± 0.06	2.36 ± 0.21^***^	1.40 ± 0.08^###^
Heart weight (mg)	143.66 ± 2.75	173.55 ± 7.77^*^	133.31 ± 4.18^#^
Epididymal fat weight (g)	0.72 ± 0.08	2.49 ± 0.16^***^	1.79 ± 0.21^#^
Heart weight to body weight (mg/g)	4.63 ± 0.12	3.34 ± 0.11^***^	3.8 ± 0.12
Body weight to tibia length ratio (g/mm)	1.70 ± 0.05	2.74 ± 0.04^***^	2.04 ± 0.04^###^
Heart weight to tibia length ratio (mg/mm)	7.82 ± 0.19	9.75 ± 0.34^***^	7.42 ± 0.19^###^
**Hormone levels**			
	**ND** (***n* = 5)**	**HFHC** (***n* = 5)**	**HFHC + PFD** (***n* = 5)**
Resistin (pg/mL)	55,051.6 ± 4,168.4	89,032.2 ± 10,975.5^*^	85,795.1 ± 5,184.5
Insulin (pg/mL)	5,754 ± 449.1	18,030 ± 2,749^***^	9,192 ± 968.8^##^
Glucagon (pg/mL)	2,368.1 ± 592.7	5,184.4 ± 584.8^*^	1,601.3 ± 624^##^
Leptin (pg/mL)	1,470 ± 192.6	14,678.8 ± 2,094^***^	4,475.7 ± 846.4^###^
PAI-1 (pg/mL)	715.3 ± 111.3	2,424 ± 301.8^***^	1,058.4 ± 152.9^##^
GIP (pg/mL)	135.5 ± 14.31	193.3 ± 54.61	135.4 ± 15.53
**Serum liver enzymes**			
	**ND** (***n* = 5)**	**HFHC** (***n* = 5)**	**HFHC + PFD** (***n* = 5)**
ALT (U/L)	35.2 ± 5.46	69.6 ± 7.98^**^	46.40 ± 1.20^#^
AST (U/L)	84 ± 3.50	139.6 ± 18.83^**^	98.2 ± 1.68^#^

Body weight, organ weights, hypertrophy indices, and serum biomarker levels in the experimental mouse groups. Data are expressed as mean ± standard error of the mean (SEM). One-way ANOVA followed by Tukey’s *post hoc* test. ^*^P < 0.05, ^**^P < 0.01, ^***^P < 0.001 vs. ND; ^#^*P* < 0.05, ^##^*P* < 0.01, ^###^*P* < 0.001 vs. HFHC

Caloric intake was significantly higher in both HFHC mice and those treated with PFD compared to the ND group (Fig. 1b, c). HFHC mice developed hyperglycemia and impaired insulin sensitivity compared to ND mice, respectively (*P* < 0.001). Notably, PFD intervention in HFHC mice significantly ameliorated insulin sensitivity (*P* < 0.001 vs. HFHC), which was analyzed and quantified using the AUC method (Fig. 1d-f).

Obese MASH mice showed significant (*P* < 0.001) elevation in serum triglycerides, total cholesterol, LDL-C, and VLDL-C compared with the ND group, whereas PFD reduced these levels of triglycerides (*P* < 0.01), total cholesterol (*P* < 0.001), LDL-C (*P* < 0.001), and VLDL-C (*P* < 0.001), compared with the HFHC animals (Fig. 1g-j).

Table 1 shows the end-point anthropometric parameters. HFHC animals gained significantly more body weight and organ weights (including liver, heart, and epididymal fat) compared with those from ND mice. However, HFHC animals receiving PFD exhibited a significant reduction in final body weight (*P* < 0.001), as well as liver (*P* < 0.001), heart (*P* < 0.05) and epididymal fat (*P* < 0.05) when compared to the untreated HFHC group. As expected, HFHC mice showed an increased HW/BW ratio, and HW/TL ratio (*P* < 0.001), both of which were statistically significantly restored by PFD (Table 1). The intake of a Western diet significantly increased the circulating levels of metabolic hormones, including leptin (*P* < 0.001), insulin (*P* < 0.001), resistin (*P* < 0.05), glucagon (*P* < 0.05), and PAI-1 (*P* < 0.001). PFD treatment significantly reduced these hormone levels, including leptin (*P* < 0.001), insulin (*P* < 0.01), glucagon (*P* < 0.01), and PAI-1 (*P* < 0.01). However, resistin and GIP levels showed a tendency to decrease in mice with PFD, but these changes were not significant. High levels of serum liver enzymes, specifically ALT (*P* < 0.01) and AST (*P* < 0.01) were found in obese mice, but PFD treatment in mice fed an HFHC diet significantly reduced ALT (*P* < 0.05) and AST (*P* < 0.05) biochemical activities (Table 1).

### PFD decreases histological damage to the liver in obese MASH mice

As expected, histological analysis of the HFHC mice fed for 16 weeks showed statistically significant liver steatosis (encompassing both microsteatosis and macrosteatosis), hepatocyte ballooning, inflammatory changes, and fibrosis. Concurrently, PFD in obese mice significantly prevented these histological alterations, reducing the severity of steatosis (*P* < 0.001), the number of inflammation foci (*P* < 0.001), and the fibrosis area percentage (*P* < 0.001). Furthermore, the increased adipocyte area observed in obese animals (*P* < 0.001) was markedly reduced after PFD intervention in obese mice (*P* < 0.001) (Supplementary Fig. S2).

### PFD improves cardiac hypertrophy, inflammation, and fibrosis induced by obesity

**Fig. 2:**
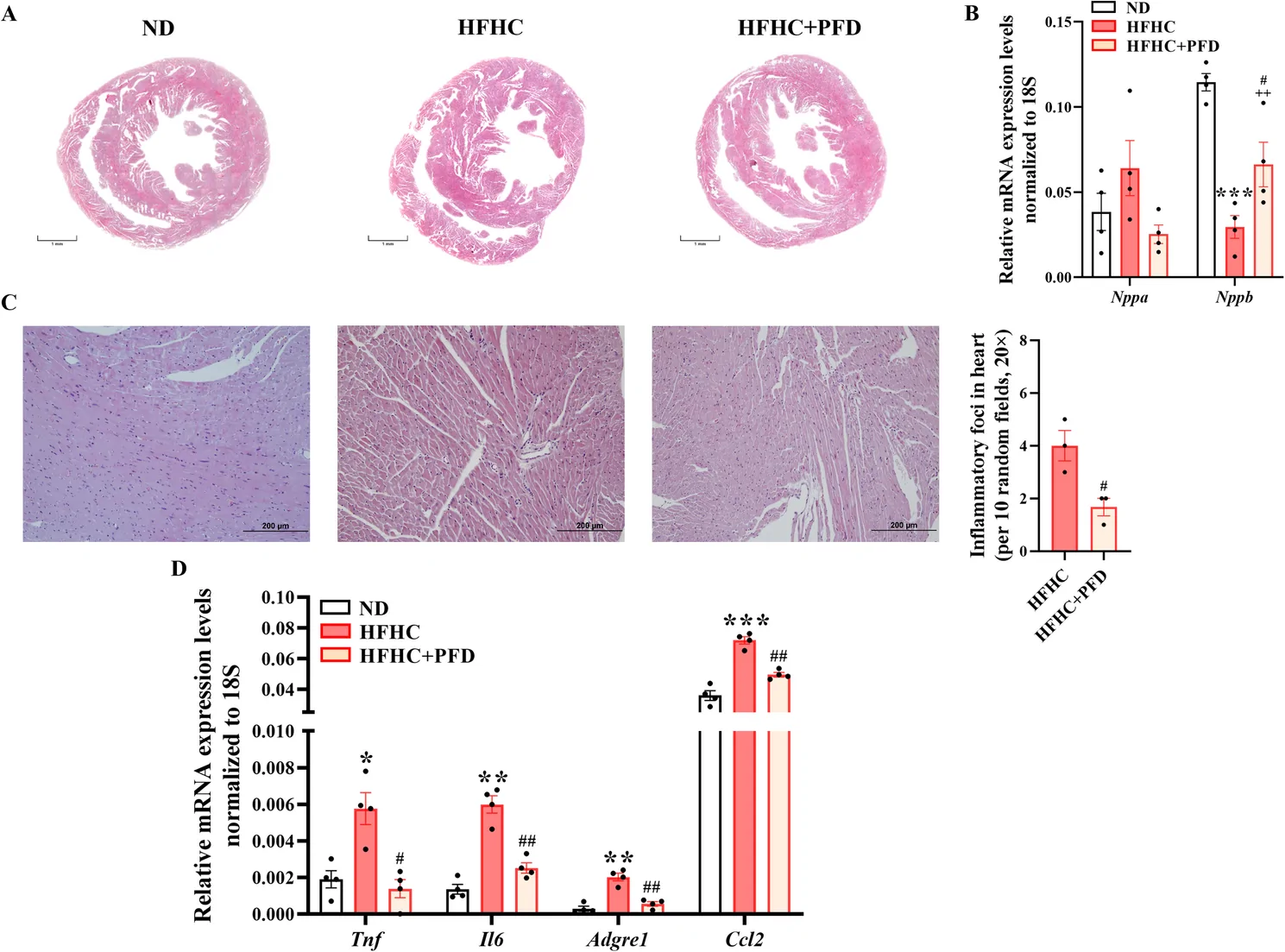
PFD improves cardiac hypertrophy and cardiac inflammatory markers in obese mice. (**A**) Micrographs of the ventricular chamber tissue sections. (**B**) Relative mRNA expression levels of hypertrophy markers (*Nppa*, *Nppb*). (**C**) Number of inflammatory foci (Student’s t-test, *n* = 3 per group). (**D**) Relative mRNA expression levels of inflammatory markers (*Tnf*, *Il6*, *Adgre1*, *Ccl2*), *n* = 4 per group. One-way ANOVA followed by Tukey’s post hoc test. Data are expressed as mean ± SEM. *P < 0.05, **P < 0.01, ***P < 0.001 vs. ND; ^#^*P* < 0.05, ^##^*P* < 0.01 vs. HFHC; ^++^*P* < 0.01 vs. ND

Figure 2a shows the histological examinations in the ventricular chamber tissue sections from mice. As shown in Fig. 2b, transcript levels of the hypertrophy biomarker *Nppa* mRNA levels tended to increase, whereas *Nppb* mRNA levels (*P* < 0.001) were reduced in obese mice compared to ND mice. Conversely, in obese mice treated with PFD, there were significantly lower *Nppa* mRNA levels (*P* < 0.05) and significantly higher *Nppb* mRNA levels (*P* < 0.05) than in those from HFHC animals. We also analyzed the presence of neutrophilic inflammatory foci in the ventricular tissue of mice, and the inflammatory foci induced in HFHC mice were significantly (*P* < 0.05) prevented by PFD treatment (Fig. 2c). In obese mice, these histopathological observations were accompanied by an increase in the mRNA levels of *Tnf* (*P* < 0.05), *Il6* (*P* < 0.01), *Adgre1* (*P* < 0.01), and *Ccl2* (*P* < 0.001). However, PFD significantly prevented the increased mRNA levels of *Tnf* (*P* < 0.05), *Il6* (*P* < 0.01), *Adgre1* (*P* < 0.01), and *Ccl2* (*P* < 0.001) (Fig. 2d).

**Fig. 3:**
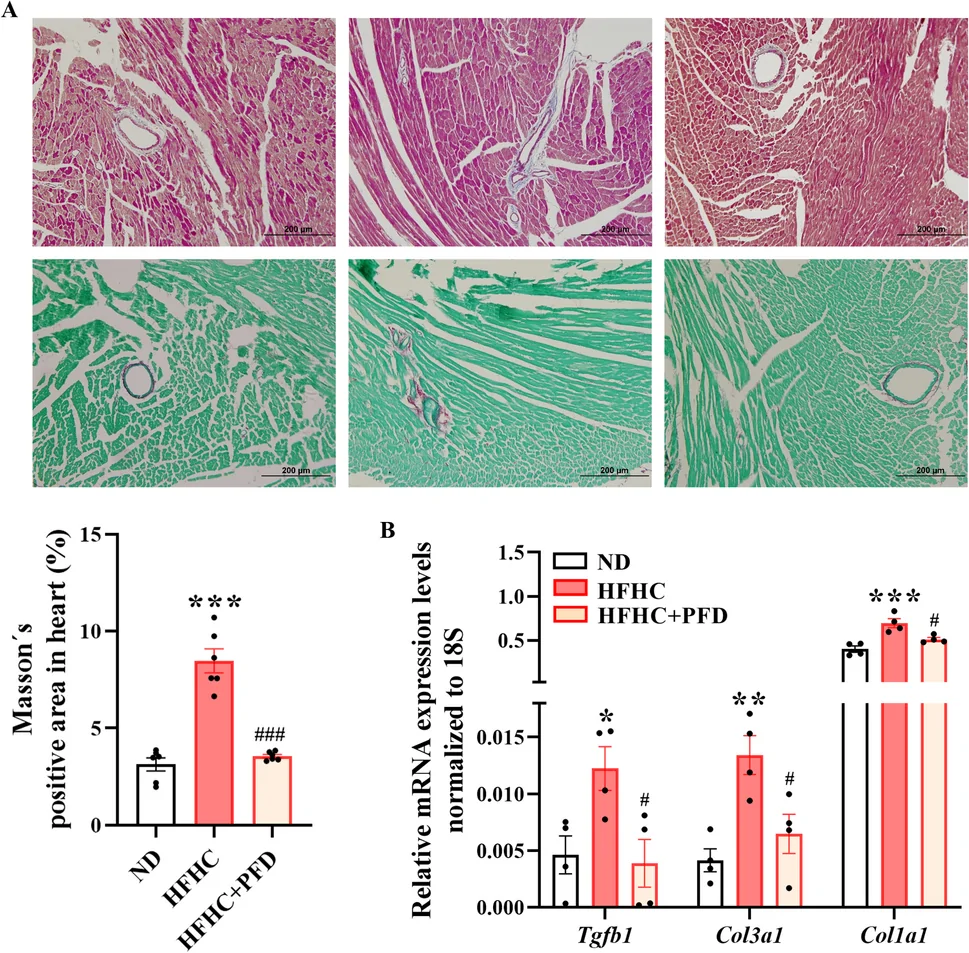
Cardiac fibrosis is prevented by PFD. (**A**) Cardiac fibrosis was assessed with Masson’s trichrome staining (*n* = 6 per group). (**B**) Relative mRNA expression levels of fibrotic markers: *Tgfb1*, *Col3a1, *and *Col1a1* (*n* = 4 per group). Data are expressed as mean ± SEM. One-way ANOVA followed by Tukey’s post hoc test. *P < 0.05, **P < 0.01, ***P < 0.001 vs. ND; ^#^*P* < 0.05, ^###^*P* < 0.001 vs. HFHC

In order to examine the effect of the Western diet on extracellular matrix fiber accumulation in cardiac tissues, we analyzed ventricular tissue from mouse groups through Masson’s trichrome and Picrosirius red staining. Ventricular tissues from obese mice showed a significant (*P* < 0.001) fibrotic area (7%), but PFD intervention significantly (*P* < 0.001) reduced this pathological fibrotic parameter to approximately 3% (Fig. 3a). Furthermore, we evaluated changes in the mRNA expression levels of genes related to fibrosis in ventricular tissue, which were statistically significantly higher in obese mice, such as *Tgfb1*, *Col1a1*, and *Col3a1*, than those in obese mice receiving PFD (Fig. 3b).

### PFD restores cardiac autophagy in an obesity-induced mouse MASH model by p-AMPK activation

**Fig. 4:**
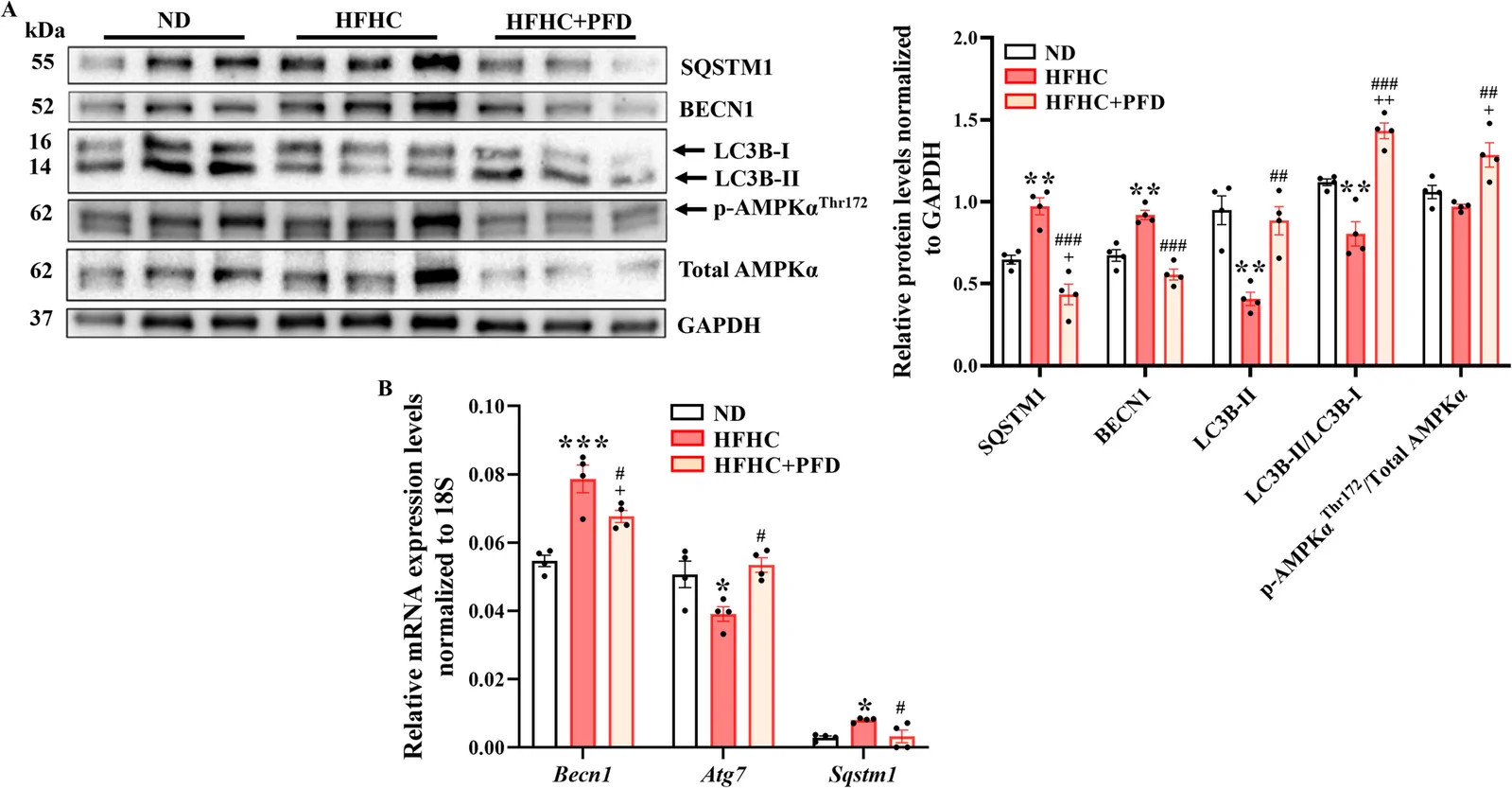
Evaluation of cardiac autophagy-related markers in mice. (**A**) Western blotting image and relative protein expression levels of SQSTM1, BECN1, LC3B-II/LC3B-I ratio, and phosphorylated p-AMPKα (Thr172) normalized to total AMPKα and GAPDH (*n* = 4 per group). (**B**) Relative mRNA expression levels: *Becn1*, *Atg7*, *and Sqstm1* (*n* = 4 per group). Data are expressed as mean ± SEM. One-way ANOVA followed by Tukey’s post hoc test. *P < 0.05, ***P < 0.001 vs. ND; ^#^*P* < 0.05, ^##^*P* < 0.01 vs. HFHC

Autophagy is decreased in obesity and diabetes; thus, we evaluated the potential cardiovascular effects of PFD on this important cellular process. The most important autophagy markers in ventricular tissue of mice were evaluated. As expected, ventricular tissues from obese mice showed a severe impairment of downstream autophagic flux rather than a simple failure in initiation. This specific disruption was evidenced by the increase in protein levels of SQSTM1, also known as p62 (*P* < 0.01), and BECN1 (*P* < 0.05), coupled with the reduction in protein levels of LC3B-II/GAPDH (*P* < 0.05), the LC3B-II/LC3B-I ratio (*P* < 0.01) and the active form p-AMPK (Thr172) showing a tendency to decrease, respectively, quantified across *n* = 4 independent biological replicates. This concomitant accumulation of both the autophagosome initiation marker (BECN1) and the cargo adapter (SQSTM1) explicitly indicates a late-stage blockade in autophagic degradation under metabolic stress. Noteworthy, these autophagy activation markers in obese mice were restored markedly following PFD treatment, including SQSTM1 (*P* < 0.001), BECN1 (*P* < 0.05), LC3B-II/GAPDH (*P* < 0.05), the LC3B-II/LC3B-I ratio (*P* < 0.001) and p-AMPK (*P* < 0.05) (Fig. 4a), demonstrating that PFD reactivates cardiac autophagy. Of note, p-AMPK (Thr172) is a master metabolic sensor that restores cellular energy balance during stress. These findings were supported by the corresponding transcript profiles. In obese mice, we found a significant increase in *Sqstm1* (*P* < 0.05) and *Becn1* (*P* < 0.001), and a decrease in *Atg7* (*P* < 0.01) compared to ND mice, while in HFHC mice with PFD, the mRNA expression levels were significantly reduced for *Sqstm1* (*P* < 0.05) and *Becn1* (*P* < 0.05), and elevated for *Atg7* (*P* < 0.001) compared to HFHC animals (Fig. 4b). Together, these findings demonstrate that autophagy suppression in ventricular tissue in obese mice is restored by PFD.

### Determination of the cytotoxicity of compounds and lipid droplets in H9c2 cells

**Fig. 5:**
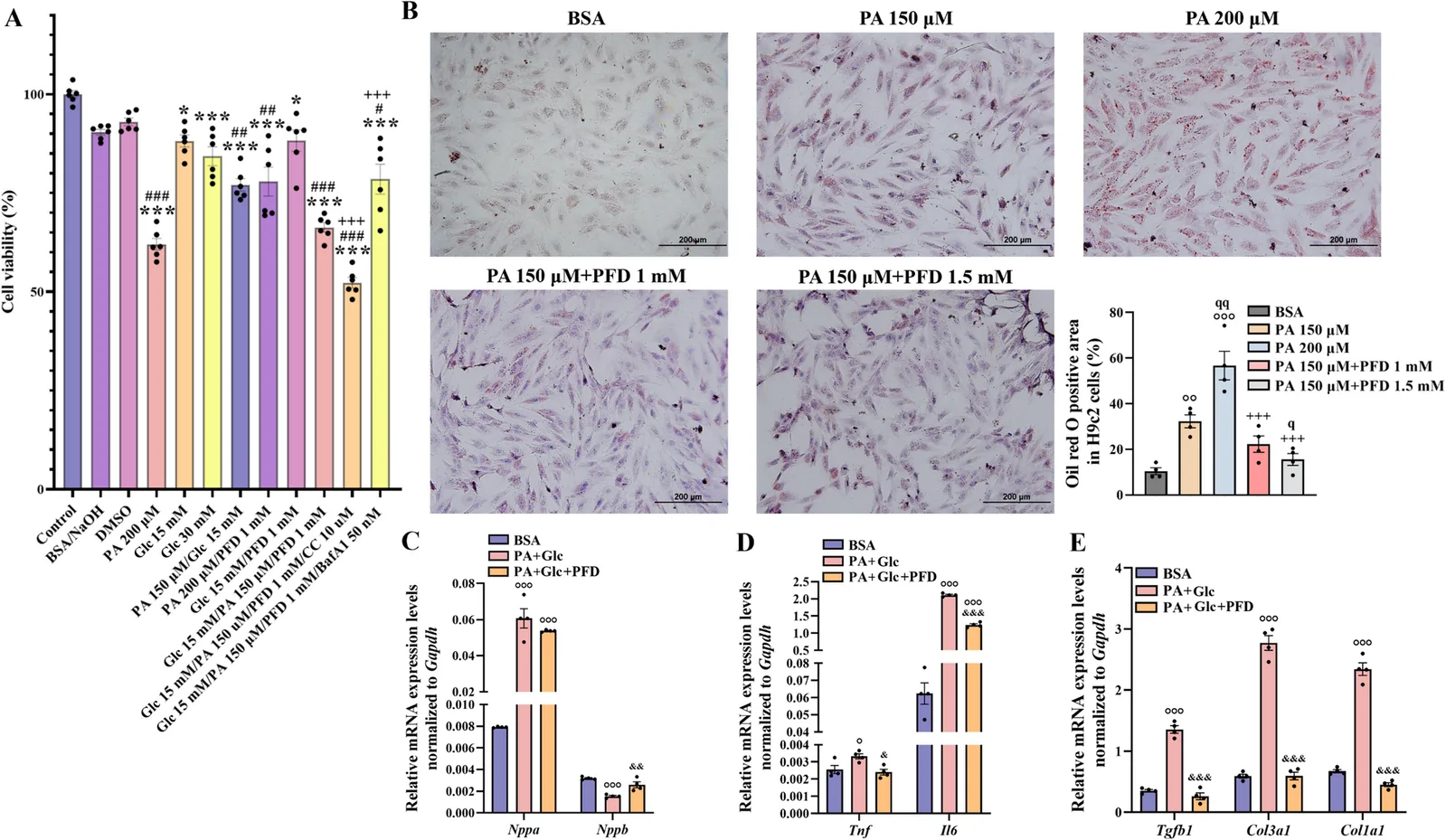
Cell viability by MTT assay in H9c2 cardiomyoblasts. (**A**) Concentrations of compounds chosen for experiments in H9c2 cells. (**B**) Representative micrographs showing intracellular lipid droplets (ORO). Scale bar = 200 μm. (**C**) Quantification of Oil Red O-positive stained area percentage (*n* = 4 fields per condition. (**D**-**F**) Relative mRNA expression levels of *Nppa, Nppb, Tnf, Il6, Tgfb1, Col3a1,* and *Col1a1* normalized to *Gapdh* in H9c2 cells treated with bovine serum albumin (BSA), palmitic acid (PA), glucose (Glc), or pirfenidone (PFD), compound C (CC), and bafilomycin A1 (BafA1) (*n* = 4). Data are expressed as mean ± SEM. One-way ANOVA followed by Tukey’s post hoc test (*n* = 6 per group). *P < 0.05, ***P < 0.001 vs. control; ^#^*P* < 0.05, ^##^*P* < 0.01, ^###^*P* < 0.001 vs. BSA/NaOH; ^+++^*P* < 0.001 vs. DMSO. °*P* < 0.05, °°°*P* < 0.001 vs. BSA; ^q^P < 0.05, ^qq^P < 0.01 vs. PA 150 µM; ^&^*P* < 0.05, ^&&^*P* < 0.01, ^&&&^*P* < 0.001 vs. PA 150 µM + Glc 15 mM

Cell viability was assessed for palmitic acid (PA) and glucose to establish a glucolipotoxicity-induced model in H9c2 cells. PA at 400 µM reached a cell viability of 50% (*P* < 0.001), while glucose at 30 mM had 76% (*P* < 0.001). Concurrently, PA at 150 µM with glucose at 30 mM showed 63% (*P* < 0.001). Interestingly, PFD at 1 mM significantly increased cell viability by around 10% (*P* < 0.05) (Supplementary Fig. S3a-c). Next, we evaluated PA at 200 µM in combination with PFD (0.3, 0.5, 1, and 1.5 mM), which showed a tendency to recover the cell viability up to 1 mM; while glucose 30 mM plus PFD 1 mM showed a cell viability of 75% (Supplementary Fig. S4a). PA at 200 µM was evaluated with compound C (CC, 10 and 50 µM) or with bafilomycin A1 (BafA1, 50 and 100 nM), showing cell viability for CC (65 and 55%) and BafA1 (67 and 55%), respectively (Supplementary Fig. S4b). According to these results, we chose to evaluate CC at 10 µM and BafA1 at 50 nM in combination with different concentrations of PA, glucose, and PFD. The cell viability results showed that CC at 10 µM had the most toxicity combined with PA at 200 µM and PFD at 0.5 or 1 mM (50%) (Supplementary Fig. S4c), while CC at 10 µM with glucose at 15 mM plus PA at 150 µM plus PFD 1 mM also exhibited 50% (Supplementary Fig. S4d). Therefore, we chose optimal concentrations for PA, glucose, PFD, CC, and BafA1 to evaluate gene expression, which is shown in Fig. 5a. Oil Red O staining revealed that PFD intervention attenuated PA-induced lipid accumulation in H9c2 cells (Fig. 5b). Quantification of the Oil Red O staining demonstrated a significant increase with PA at 150 µM and 200 µM; however, the co-administration of PA at 150 µM plus PFD at 1.5 mM significantly decreased the Oil Red O-positive area percentage (*P* < 0.05) (Fig. 5c). H9c2 cells under glucolipotoxicity had significantly higher *Nppa* mRNA levels (*P* < 0.001) and significantly lower *Nppb* mRNA levels (*P* < 0.001) compared to control cells, while PFD significantly increased *Nppb* mRNA levels (*P* < 0.01) (Fig. 5d). mRNA levels of *Tnf* and *Il6* were statistically significantly higher in H9c2 cells under glucolipotoxicity, but PFD reduced the transcript levels of these inflammation genes (Fig. 5e). mRNA levels of *Tgfb1*,* Col1a1*, and *Col3a1* were also analyzed in H9c2 cells and were significantly higher (*P* < 0.001) under glucotoxic and lipotoxic conditions, whereas PFD significantly reduced these mRNA levels (*P* < 0.001) (Fig. 5f).

### PFD restores cardiac autophagy in glucolipotoxicity-induced H9c2 cells by p-AMPK activation

**Fig. 6:**
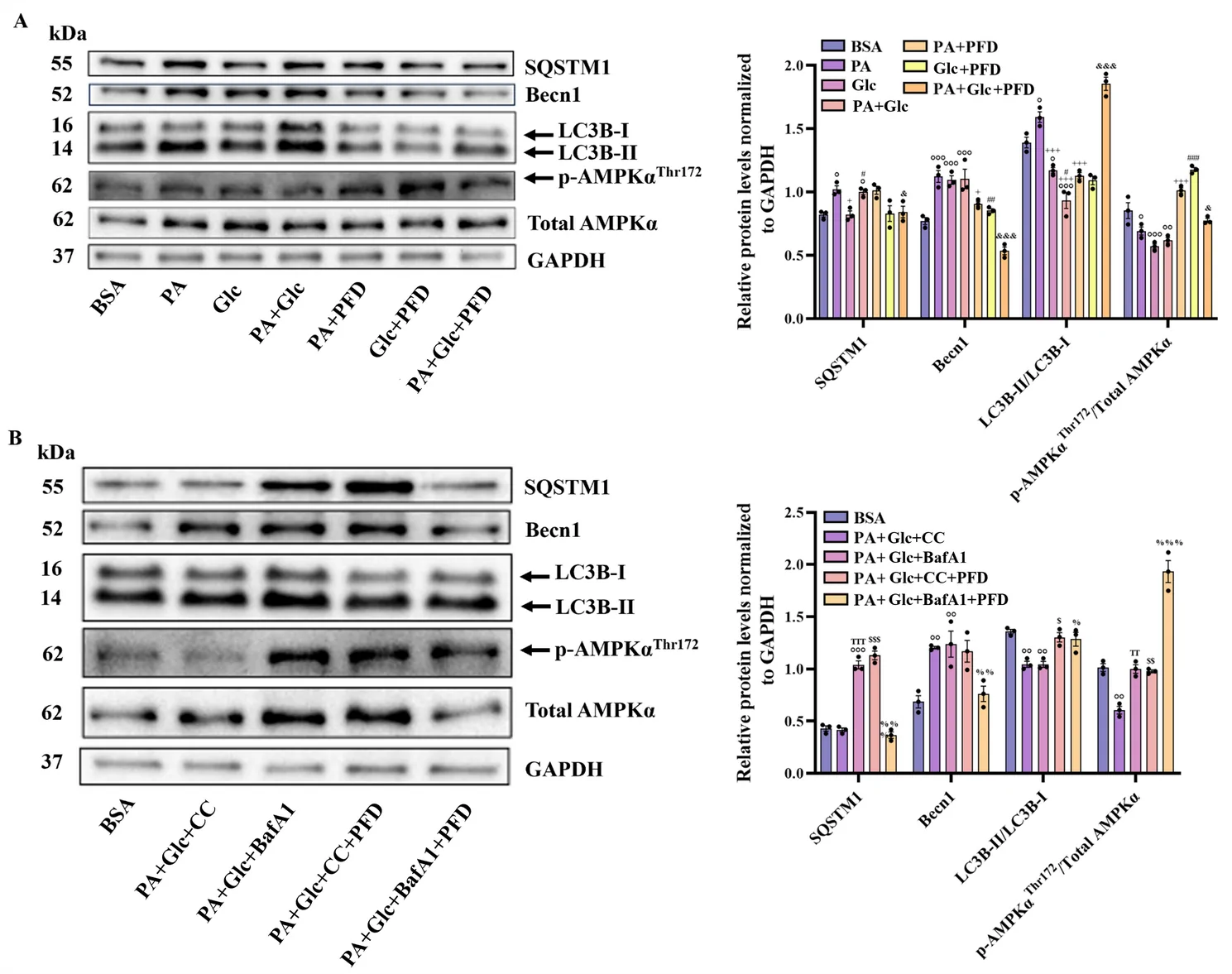
Evaluation of autophagy-related markers in H9c2 cardiomyoblasts. **A**-**B**) Western blotting image and relative protein expression levels of SQSTM1, BECLIN-1, LC3B-II/LC3B-I ratio, and p-AMPKα (Thr172) normalized to total AMPKα and GAPDH (*n* = 4 per group). **C**-**D**) Relative mRNA expression levels: *Becn1*, *Atg7*, and *Sqstm1* (*n* = 4 per group). Data are expressed as mean ± SEM. One-way ANOVA followed by Tukey’s post hoc test. °*P* < 0.05, °°*P* < 0.01, °°°*P* < 0.001 vs. control; ^+^*P* < 0.05, ^+++^*P* < 0.001 vs. PA 200 µM; ^#^*P* < 0.05, ^##^*P* < 0.01, ^###^*P* < 0.001 vs. Glc 30 mM; ^&^*P* < 0.05, ^&&&^*P* < 0.001 vs. PA 150 µM + Glc 15 mM; ^TT^*P* < 0.01, ^TTT^*P* < 0.001 vs. PA 150 µM + Glc 15 mM + CC 10 µM; ^$^*P* < 0.05, ^$$^*P* < 0.01, ^$$$^*P* < 0.001 vs. PA 150 µM + Glc 15 mM + CC 10 µM; ^%^*P* < 0.05, ^%%^*P* < 0.01, ^%%%^*P* < 0.001 vs. PA 150 µM + Glc 15 mM + BafA1 50 nM

Given the evidence of autophagy activation in obese mice following PFD intervention, we further analyzed the molecular effects using autophagy biomarkers in glucolipotoxicity-associated H9c2 cells. Western blot analysis quantified across *n* = 4 independent biological replicates showed that H9c2 cells in the presence of PA had significantly increased protein levels of SQSTM1 (*P* < 0.05), and BECN1 (*P* < 0.001), alongside an elevated LC3B-II/LC3B-I ratio (*P* < 0.001), while the active form p-AMPK (*P* < 0.05) was decreased (Fig. 6a), indicating an upregulation of autophagy in response to cellular stress. Conversely, H9c2 cells treated with glucose showed an increase for BECN1 (*P* < 0.001), accompanied by a reduction in the LC3B-II/LC3B-I ratio (*P* < 0.001), and p-AMPK (*P* < 0.001) compared to the control, which indicates the inhibition of autophagosomal maturation. Subsequently, we analyzed the combined effects of both PA and glucose in H9c2 cells, simulating the metabolic stress of obese mice fed a Western diet; under these conditions, SQSTM1 (*P* < 0.05) and BECN1 (*P* < 0.001) were increased, while the LC3B-II/LC3B-I ratio (*P* < 0.001) and p-AMPK (*P* < 0.01) were reduced (Fig. 6a), indicating a profound inhibition of autophagic flux. When H9c2 cells were treated with PA + PFD, protein levels of BECN1 (*P* < 0.05) and the LC3B-II/LC3B-I ratio (*P* < 0.001) were reduced compared to cells exposed only to PA, while p-AMPK (*P* < 0.01) was significantly higher than in cells with PA. Meanwhile, glucose with PFD in H9c2 cells showed a significant reduction in BECN1 (*P* < 0.01) and an increase in p-AMPK (*P* < 0.001). Similar to the obese mice results, H9c2 cells treated with PA+glucose + PFD showed lower protein levels of SQSTM1 (*P* < 0.05) and BECN1 (*P* < 0.001). Noteworthy, higher levels of the LC3B-II/LC3B-I ratio (*P* < 0.001) and p-AMPK (*P* < 0.05) were found compared to cells exposed to PA+glucose, suggesting the effective reactivation of the autophagic pathway (Fig. 6a).

Bafilomycin A1 (BafA1) is known as an inhibitor of the late phase of autophagy by preventing the formation of autophagolysosomes (in the phagosome-lysosome fusion stage), whereas compound C (CC) is an AMPK inhibitor. Therefore, both were used to assess the role of PFD and p-AMPK in the autophagy flux of H9c2 cells. All H9c2 cell variables were treated with PA and glucose; when these cells were treated additionally with CC, they showed increased protein levels of BECN1 (*P* < 0.01) and reduced levels of the LC3B-II/LC3B-I ratio (*P* < 0.01) and p-AMPK (*P* < 0.01), suggesting autophagy inhibition (Fig. 6b). Meanwhile, the presence of BafA1 increased SQSTM1 (*P* < 0.001) and BECN1 (*P* < 0.01) and reduced the LC3B-II/LC3B-I ratio (*P* < 0.01) compared to PA and glucose. This BafA1-mediated accumulation profiles successfully recapitulated the late-stage autophagic flux blockade observed in our vehicle-treated MASH models. Although H9c2 cells were grown in the presence of PA, glucose, CC, and PFD, they showed a significant increase in SQSTM1 (*P* < 0.001), in the LC3B-II/LC3B-I ratio (*P* < 0.05), and in p-AMPK (*P* < 0.01), suggesting that PFD through p-AMPK induction promotes autophagy. Interestingly, H9c2 cells in the presence of PA, glucose, BafA1, and PFD exhibited a reduction of SQSTM1 (*P* < 0.001) and BECN1 (*P* < 0.01), and an increase of the LC3B-II/LC3B-I ratio (*P* < 0.05), including markedly higher levels of p-AMPK (*P* < 0.001) than those levels expressed in cells with PA+glucose+BafA1, suggesting that PFD overcomes the lipotoxicity-induced degradation bottleneck to reactivate functional autophagic flux (Fig. 6b).

The mRNA expression levels of *Sqstm1* and *Becn1* were similar to their protein accumulation profiles (Fig. 6c, d). In H9c2 cells with PA, mRNA expression levels of *Atg7* were significantly increased compared to control (*P* < 0.01), while these levels were statistically significantly reduced in the presence of glucose, PA+glucose, and PA + PFD compared to PA. In H9c2 cells with PA+glucose + PFD, mRNA expression levels of *Atg7* were significantly increased compared to H9c2 cells with PA+glucose (*P* < 0.001) (Fig. 6c). In the presence of PA+glucose with CC or BafA1, mRNA expression levels of *Atg7* were significantly decreased compared to the control (*P* < 0.001). PA+glucose + CC+PFD significantly increased the mRNA expression levels of *Atg7* compared to PA+glucose + CC (*P* < 0.01); the presence of PA+glucose+BafA1 + PFD also increased their levels compared to PA+glucose+BafA1 (*P* < 0.05) (Fig. 6d).

### HFHC diet and PFD-induced regulation of ventricular gene expression

**Fig. 7:**
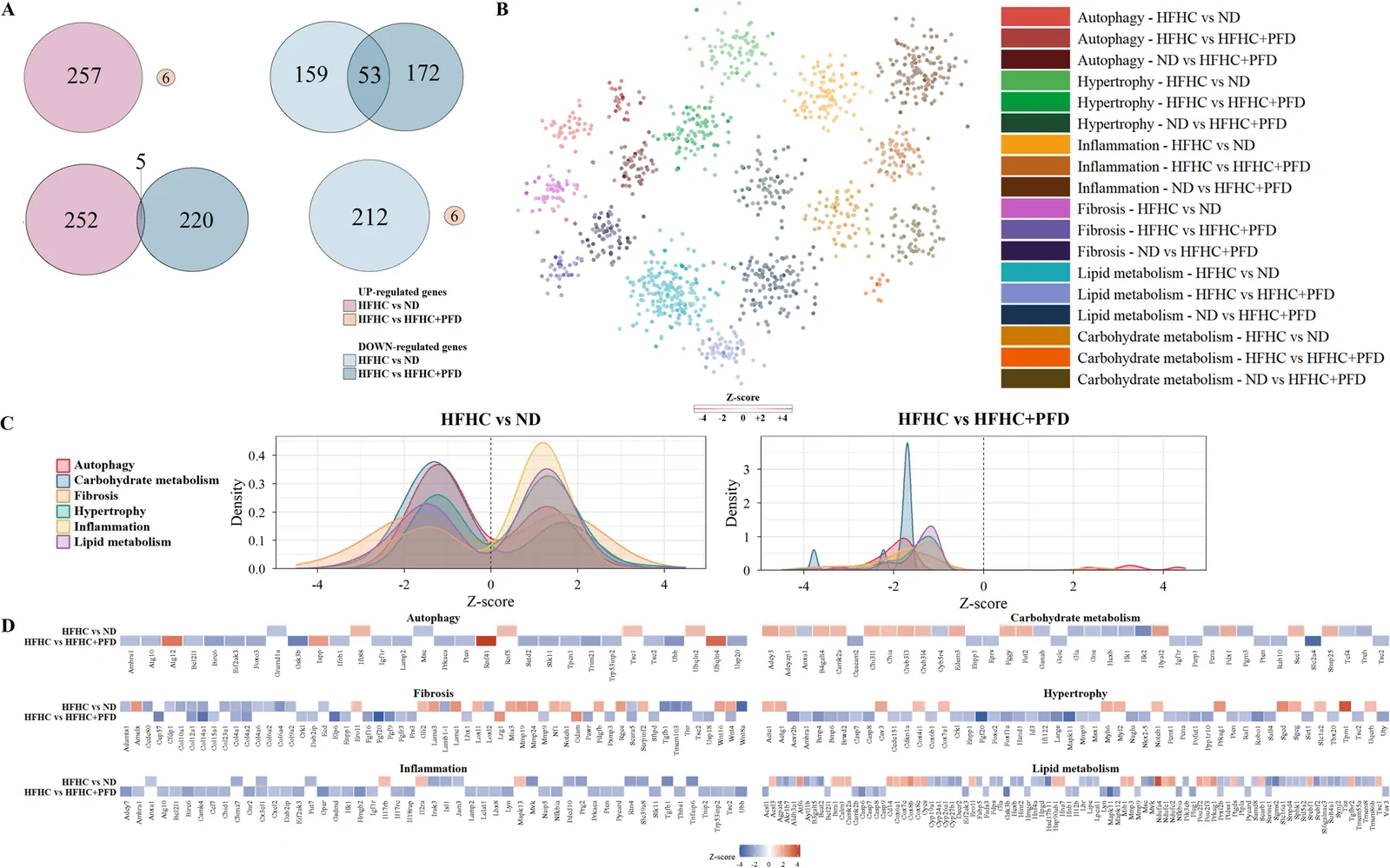
Differentially expressed genes in ventricular tissue induced by HFHC diet and PFD. (**A**) Venn diagram analysis comparing gene expression identified among the mouse groups. (**B**) Biological processes-structured visualization of differentially expressed genes. The color intensity corresponds to Z-score magnitude. (**C**) Kernel density plots showing the Z-score distribution of gene expression. Panels A-C include genes with Z-score ≥ + 1.5 (up-regulated) or Z-score ≤ -1.5 (down-regulated). (**D**) Heatmaps displaying gene-specific Z-scores (Z-score ≥ + 1.5 or Z-score ≤ -1.5), where rows represent comparisons and columns represent individual genes

Microarray analysis was performed using RNA extracted from the ventricular tissue of mice. Venn diagrams depict the distribution of regulated genes according to selected biological processes (autophagy, carbohydrate and lipid metabolism, fibrosis, hypertrophy, and inflammation) (Fig. 7a). Figure 7b shows DEGs in an AD2 space designed to visually group genes by biological process and experimental comparison. Transcriptomic changes induced by the HFHC diet and those caused by the presence of PFD were distributed according to biological processes. Kernel density plots display the distribution of Z-scores for genes within each biological process (Fig. 7c). To examine relative gene expression patterns between groups, heatmaps were generated to compare differences between mouse groups. As shown in Fig. 7d, each process contains genes with marked changes associated with HFHC, and in the comparison between HFHC and HFHC + PFD, some gene subsets show patterns suggesting attenuation or directional changes, while other genes retain signals consistent with persistent alterations.

To complement this global transcriptomic characterization, a comprehensive functional enrichment analysis was performed using DAVID Bioinformatics Resources. Among the differentially regulated transcripts, Gene Ontology (GO) analysis identified highly significant biological processes targeted by the PFD intervention, including the regulation of autophagic flux (GO:0006914, *P* = 3.69E-07), regulation of cardiac muscle hypertrophy (GO:0010163, *P* = 9.94E-15), and cardiac muscle tissue development (GO:0048738, *P* = 2.44E-09). Conversely, the untreated HFHC diet triggered a significant transcriptomic enrichment in inflammatory response pathways (GO:0050729, *P* = 2.04E-16) and extracellular matrix organization (GO:0030198, *P* = 2.72E-06). Furthermore, the top differentially expressed genes (DEGs) displaying the most pronounced fold-changes and directional recovery under PFD treatment were systematically prioritized. A detailed list containing these top 20 altered transcripts, their corresponding fold-change/Z-score values, specific GO term annotations, and enriched metabolic pathways is fully documented in Supplemental Table S3.

## Discussion

The Western diet triggers obesity, increased adiposity, systemic insulin resistance, elevated fasting glucose, and MASH characterized by inflammation and fibrosis, thereby accelerating the risk of cardiovascular disease in C57BL/6J mice [6, 7, 20, 21]. Mechanistically, insulin resistance impairs lipoprotein lipase activity (a major mediator of VLDL clearance) and drives chronic inflammation via the upregulation of TNF-α and IL-1β, hallmarks often observed in obesity and diabetes. Furthermore, insulin resistance independently increases cardiovascular risk factors under diabetic conditions [2]. As expected, the obese mice exposed to the Western diet develop alterations in lipid profiles, increased ALT and AST activities, and high fasting glucose levels after intake of the HFHC diet for 16 weeks [6, 7, 20]. PFD prevented dyslipidemia, reduced ALT and AST levels, and improved insulin resistance, aligning with previous preclinical evidence [7, 19, 20, 22–25]. MASH progression further accentuates hepatic and systemic insulin resistance, promoting atherogenic dyslipidemia and the systemic release of pro-inflammatory factors, vasoactive factors, and thrombogenic molecules, thus contributing to the development of CVD [2].

The heart weight to body weight (HW/BW) ratio and heart weight to tibia length (HW/TL) ratio are used as reliable biometric indicators for cardiac hypertrophy, while an elevated body weight to tibia length (BW/TL) ratio is an established marker in diabetic mice. In our study, the HW/BW, HW/TL, and BW/TL ratios observed in obese mice with systemic insulin resistance were consistent with previous literature [7, 26, 27], but the PFD intervention restored these parameters, suggesting a significant attenuation of metabolic stress-induced cardiac injury.

Here we demonstrate that PFD prevents the metabolic hormone dysregulation in obese mice with MASH. During obesity, leptin and insulin hypersecretion occur primarily in response to adipose tissue accumulation and hyperglycemia, respectively. Although physiological levels of leptin and adiponectin promote insulin sensitivity, an excess of other hormones contributes to insulin resistance, such as resistin, glucagon, PAI-1, and GIP. Chronic hyperleptinemia is closely linked to insulin resistance and T2D, directly contributing to obesity-related complications like hepatic fibrosis, cardiac inflammation, myocardial fibrosis, and impaired cardiac metabolism [2, 3, 28]. Systemic insulin resistance, coupled with adipose tissue dysfunction, drives MASH development and accelerates hepatic fibrosis progression. MASH may increase the risk of CVD by exacerbating hepatic and systemic insulin resistance and dyslipidemia [2]. Furthermore, hyperinsulinemia-mediated overactivation of insulin signaling inhibits autophagy, and can drive cardiac hypertrophy and pathological remodeling [29]. In addition to inducing lipolysis with high serum free fatty acid, resistin increases foam cell formation within atherosclerotic lesions [3]. Resistin is associated with inflammation (directly driving cardiomyocytes), promoting secretion of TNF-α and IL-12 in macrophages [30]. Resistin induces cardiac hypertrophy and myocardial insulin resistance via the suppression of AMPK activity [31]. In obesity or diabetes, resistin promotes fibrogenesis via JAK2/STAT3 and JNK/c-Jun signaling in cardiac fibroblasts [32]. PAI-1 (pro-inflammatory adipokine) is significantly upregulated in obesity and MASH. PAI-1 promotes atherogenesis with macrophage accumulation, increasing cardiovascular risk, and its inhibition attenuates atherosclerosis in obese mice [3]. Interestingly, elevated levels of PAI-1 can predict the onset of myocardial infarction [33]. Hyperglucagonemia is associated with individuals with fatty liver disease [34]. Glucagon was associated with insulin resistance, increased ALT, dyslipidemia, and hypertension, indicating its association with cardiometabolic risk factors [35].

The excessive triglyceride accumulation in myocardium tissue (under obesity, insulin resistance, or diabetes) is highly associated with chronic inflammation, myocardial hypertrophy, and fibrosis [7, 11, 36]. Paradoxically, epidemiological studies have reported reduced circulating levels of ANP (*Nppa*) and BNP (*Nppb*) in individuals with obesity, insulin resistance, and diabetes, and these low levels may increase cardiovascular risk [37]. While ANP is predominantly secreted by the cardiomyocytes in the atria, BNP is primarily secreted by the ventricular myocardium. In this study, ventricular tissues from obese mice showed an upregulation of *Nppa* mRNA and significantly reduced *Nppb* transcript levels. Controversial results have been reported for the expression of these hypertrophy biomarkers. For instance, mice fed with only a high-fat diet with 60% fat for a year showed a generalized downregulation of natriuretic peptides [38]. In obese mice fed with high fat (45% calories from fat) for 20 weeks, there was an upregulation of *Nppa* and *Nppb* mRNA levels in the left ventricles [39]. In concordance with our results, C57BL/6J mice exposed to Western diet for 2 weeks (early hypertrophic response) expressed a tendency to increase *Nppa* mRNA and decrease *Nppb* mRNA [40]. In H9c2 cells under glucolipotoxicity, *Nppa* and *Nppb* mRNA levels were also altered. Importantly, in obese mice and H9c2 cells with glucolipotoxicity, PFD intervention reestablished these hypertrophy biomarkers.

Our obese mice developed significant ventricular inflammation with fibrosis as shown by increased inflammatory foci and the upregulation of inflammatory (*Tnf*,* Il6*,* Adgre1*, and *Ccl2*) and fibrosis (*Tgfb1*,* Col1a1*, and *Col3a1*) genes [7, 41]. PFD significantly blunted this ventricular inflammation with fibrosis, as was also reported in previous works [7, 42, 43]. mRNA levels related to inflammation with fibrosis also were reversed by PFD in H9c2 cells with glucolipotoxicity.

During obesity, autophagy is inhibited or impaired across multiple metabolic organs, such as adipose tissue, the liver, and the heart, leading to the accumulation of damaged organelles and contributing to insulin resistance and tissue dysfunction [13, 44, 45]. In our study, cardiac autophagy was significantly suppressed in the cardiac tissue of obese mice with MASH. Autophagy inhibition was shown by an increased expression of autophagy-related genes, such as *Becn1* and *Sqstm1* (p62), accompanied by reduced LC3B-II protein accumulation and a decreased LC3B-II/LC3B-I ratio. Consistently, other studies have reported similar gene expression patterns in cardiac tissue from models of obesity and other metabolic disorders [44, 45]. Functionally, BECN1 orchestrates the initiation of autophagosome biogenesis, while ATG7 and LC3 proteins modulate the maturation of the autophagosomes [45]. During active autophagy, LC3B-I is lipidated into LC3B-II through an ubiquitin-like conjugation system involving ATG7, allowing LC3B-II to associate with autophagic vesicle membranes, whose membranes are selectively targeted by SQSTM1 [46]. AMPK serves as a master of mitochondrial fatty acid oxidation and cellular energy homeostasis. AMPK, in low cellular energy levels, activates ULK1 via phosphorylation, resulting in the activation of autophagy [15]. Previously, we showed in the liver of MASH mice that PR-PFD upregulated p-AMPK [20]. In our study, PFD restored autophagy by activating p-AMPK and regulating autophagy-related genes. Similarly, metformin activates AMPK, increases insulin sensitivity, and activates autophagy in several cell types [15]. Under conditions of glucolipotoxicity in H9c2 cells, we found that PA increased SQSTM1, BECN1, and the LC3B-II/LC3B-I ratio, but p-AMPK was reduced, indicative of blocked autophagic flux. Consistent with the PA effects, Zhan et al. reported a similar accumulation of SQSTM1 (p62) and an elevated LC3B-II/LC3B-I ratio expression [47]. PFD successfully activates autophagy by restoring the expression of these genes. Notably, adding PA+glucose + CC+PFD or PA+glucose+BafA1 + PFD increased the LC3B-II/LC3B-I ratio and p-AMPK, providing additional support for the involvement of late-stage autophagic processing and AMPK signaling. Similar accumulation dynamics for SQSTM1 and the LC3B-II/LC3B-I ratio expression have been observed in H9c2 cells exposed to PA+BafA1 [44]. From a mechanistic standpoint, our observation that SQSTM1 and BECN1 simultaneously accumulate while the LC3B-II/LC3B-I ratio decreases under lipotoxic stress requires a careful distinction between autophagic initiation and functional degradation flux. While elevated BECN1 implies an initial cellular attempt to orchestrate autophagosome biogenesis in response to glucolipotoxicity, the parallel accumulation of the cargo degradation substrate SQSTM1 confirms that these vesicles are not being properly cleared, highlighting a critical downstream degradative bottleneck. By utilizing Bafilomycin A1 (BafA1), a strict pharmacological blocker of autophagosome-lysosome fusion, we confirmed this metabolic dynamic. Crucially, when PFD was co-administered with PA, glucose, and BafA1, it successfully promoted p-AMPK activation and altered the protein accumulation kinetics, demonstrating that pirfenidone does not merely amplify early initiation markers but actively drives the completion of the autophagic pathway by relieving the degradative clearance blockade. Importantly, this molecular pattern should be interpreted with caution because BECN1 is primarily associated with autophagosome biogenesis, whereas the LC3B-II/LC3B-I ratio reflects steady-state LC3 lipidation and autophagosome-associated abundance. Moreover, since SQSTM1 accumulation may reflect either inefficient autophagic degradation or transcriptional upregulation, these static markers do not independently demonstrate absolute autophagic flux activation or inhibition without comprehensive tracking [48]. A previous study done in H9c2 cells with 33 mM glucose for 24 h showed inhibition of autophagy (reduced LC3B-II/LC3B-I, increased SQSTM1) [49]. However, another study that used the same glucose concentration for 48 h reported activation of autophagy [50]. These inconsistencies in results could be related to variations in autophagic flux at different stages of disease progression. We also found autophagy inhibition after 24 h of exposure in our experiments. Of course, further investigation is still needed to fully elucidate how autophagy kinetics change during the development of diabetic cardiomyopathy.

By examining approximately 22,000 gene transcripts, as illustrated in Fig. 7, it is clear that there are differentially expressed genes (DEGs) in the three groups. To provide enhanced transcriptomic depth, these DEGs were systematically prioritized based on their fold-change magnitude and functional annotation, as detailed in Supplemental Table S3. Our functional enrichment analysis via DAVID resources demonstrated that these highly altered transcripts were primarily associated with crucial biological processes underwriting the phenotype, such as autophagic flux regulation, lipid metabolism, and inflammatory cascades. Although additional secondary signaling pathways were not extensively explored in this specific microarray layout, the identification of these top-scoring DEGs provides a robust molecular footprint of PFD’s cardioprotective effects. Comparatively, a recent cardiac transcriptome analysis of diet-induced obesity by Men et al. identified a discrete set of 184 DEGs enriched for nutrient-responsive regulators and glucose metabolism dysregulation (such as *Nr4a1* upregulation) in response to a high-fat diet [51]. In alignment with their findings, our transcriptomic data underscore that chronic exposure to a Western diet induces a profound disruption in the ventricular expression profile related to cardiometabolic and homeostatic control, a pathological shift that was effectively counteracted and normalized by the standard PFD intervention.

### Limitations

**Fig. 8:**
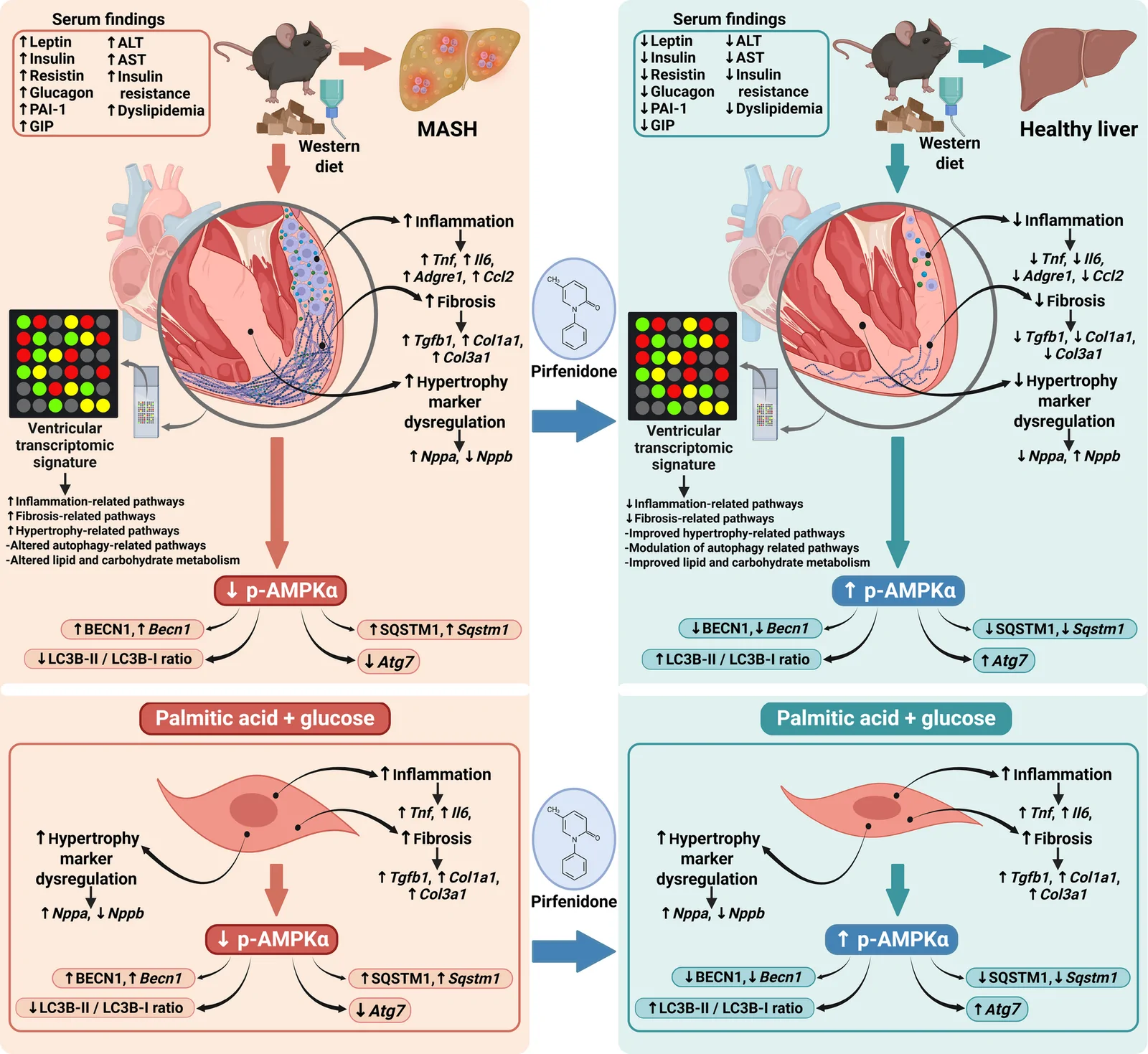
Schematic summary of the cardioprotective mechanisms of pirfenidone in obesity-associated MASH. The left panel represents the dysregulation of systemic hormone levels, alongside the induction of cardiac inflammation, hypertrophy, and fibrosis markers, including the ventricular microarray signature. Conversely, the right panel illustrates the therapeutic efficacy of PFD in reversing these pathological processes and normalizing the ventricular microarray signature profile

A limitation of the present study is that autophagic flux was not dynamically assessed in vivo. Although the evaluation of SQSTM1, BECN1, LC3B-II/LC3B-I, and p-AMPK provides highly relevant information regarding cardiomyopathy-associated signaling under steady-state conditions, these static markers cannot independently discriminate between increased autophagosome formation and reduced lysosomal clearance [48]. To overcome this in our in vitro model, BafA1 was successfully utilized to track late-stage autophagic involvement; however, advanced tandem fluorescent reporter assays, such as mRFP-GFP-LC3 or GFP-LC3-RFP-LC3ΔG constructs, were not performed. Future directions utilizing in vivo lysosomal inhibitors, cell-specific genetic models, or double-transgenic autophagy reporter mice will be valuable to further dissect the precise kinetics of PFD-mediated autophagic flux restoration in diabetic cardiomyopathy [52]. Finally, the comprehensive molecular and transcriptomic mechanisms underwriting the cardioprotective effects of PFD are schematically summarized in Fig. 8.

## Conclusion

In summary, our findings demonstrate that obesity-associated MASH triggers severe metabolic hormone dysregulation, thereby accelerating the pathological progression of cardiomyopathy. The cardiac tissue of obese mice exhibits a profound inhibition of autophagy, driven by reduced p-AMPK signaling, a molecular mechanism recapitulated in H9c2 cardiomyocytes exposed to glucolipotoxicity. Thus, suppressed AMPK activity and the consequent impairment of cardiac autophagy are key players in the progression of obesity-associated cardiomyopathy. Crucially, standard PFD prevents this autophagy inhibition by stimulating p-AMPK activity and enhancing autophagic flux. Furthermore, microarray analysis revealed distinct transcriptomic shifts, highlighting the protective molecular footprint of PFD in the cardiac tissue. Taken together, our data suggest that PFD holds significant therapeutic potential for attenuating cardiovascular complications in obesity-associated MASH.

## Supplementary Information

Below is the link to the electronic supplementary material.

Supplementary Material 1

Supplementary Material 2: Supplementary Fig. S1. Representative macroscopic appearance of mice. **A**) Representative dorsal views and abdominal cavity exposure of mice. **B**) Representative images of livers, hearts, and tibias from mice

Supplementary Material 3: Supplementary Fig. S2. PFD prevented histopathological alterations in HFHC mice. **A**) Hepatic steatotic area (Mann–Whitney *U* test, *n* = 25 per group). **B**) Number of inflammatory foci in liver sections (Student’s t-test, *n* = 7 per group). **C**) Hepatic fibrosis was quantified as the percentage of Masson’s trichrome-positive area (Kruskal–Wallis test followed by Dunn’s post hoc test, *n* = 10 per group). **D**) Adipocyte size analysis (One-way ANOVA followed by Tukey’s post hoc test, *n* = 15 per group). Data are expressed as mean ± SEM. ***P < 0.001 vs. ND; ^##^*P* < 0.01, ^###^*P* < 0.001 vs. HFHC

Supplementary Material 4: Supplementary Fig. S3. Effects of palmitic acid, glucose, and pirfenidone on H9c2 cell viability. **A**-**C**) MTT assay in H9c2 cells with increasing concentrations of palmitic acid (PA), glucose (Glc), and PA+Glc, and pirfenidone (PFD). Data are expressed as mean ± SEM. One-way ANOVA followed by Tukey’s post hoc test (*n* = 6 per group). *P < 0.05, ***P < 0.001 vs. control; ^##^*P* < 0.01, ^###^*P* < 0.001 vs. BSA/NaOH

Supplementary Material 5: Supplementary Fig. S4. Viability of H9c2 cells by MTT assays under glucolipotoxicity. **A**) Palmitic acid (PA) and pirfenidone (PFD), or glucose (Glc) and PFD. **B**) PA in combination with Compound C (CC) or bafilomycin A1 (BafA1). **C**) Combined treatments of PA+PFD with CC or BafA1, and PA+Glc+PFD. **D**) Glc+PA+PFD with CC or BafA1. Data are expressed as mean ± SEM. One-way ANOVA followed by Tukey’s post hoc test (*n* = 6 per group). *P < 0.05, ***P < 0.001 vs. Control; ^##^*P* < 0.01, ^###^*P* < 0.001 vs. BSA/NaOH; ^+++^P < 0.001 vs. DMSO

Supplementary Material 6

Supplementary Material 7

## Data Availability

Not applicable.
