# Supplementary material for: Pirfenidone restores metabolic hormones and cardiac autophagy via p-AMPK in MASH
Source: J Transl Med. 2026 Aug 19;24:1077. doi: 10.1186/s12967-026-08828-1 (PMC13495469; doi:10.1186/s12967-026-08828-1)
Supplement: Supplementary file 1 [file 12967_2026_8828_MOESM6_ESM.docx]

**Article title: Pirfenidone restores metabolic hormones and cardiac autophagy via p-AMPK in MASH**

**Journal:** **Journal of Translational Medicine**

**Authors:** Daniel López-Cifuentes ^1,2^, Ana Sandoval-Rodríguez ^1^, Rebeca Escutia-Gutiérrez ^1^, Juan Armendariz-Borunda^1,3^, Jorge Gutiérrez-Cuevas^1*^

^1^University of Guadalajara, Institute for Molecular Biology in Medicine and Gene Therapy, Department of Molecular Biology and Genomics, CUCS, Jalisco, México.

^2^Doctorate in Sciences in Molecular Biology in Medicine, University Center of Health Sciences, University of Guadalajara, Guadalajara 44340, Jalisco, Mexico.

^3^Tecnologico de Monterrey, Campus Guadalajara, Jalisco, México.

*Corresponding Author

Dr. Jorge Gutiérrez Cuevas

University of Guadalajara, Institute for Molecular Biology in Medicine and Gene Therapy, Department of Molecular Biology and Genomics, CUCS, Guadalajara, Jalisco, México.

Tel: +52-331-062-2083

E-mail: gutierrezcj05@gmail.com

**Supplementary Table S1** Primer sequences and IDs used to determine relative mRNA expression levels via RT-qPCR.

| **Target gene** | **Gene name** | **Assay ID**  **(ThermoFisher scientific)** |
| --- | --- | --- |
| *Adgre1* | Adhesion G protein-coupled receptor E1 | Mm00802529_m1 |
| *Atg7* | Autophagy related 7 | Mm00512209_m1 |
| *Atg7* | Autophagy related 7 | Rn01492725_m1 |
| *Becn1* | Beclin 1, autophagy related | Mm01265461_m1 |
| *Becn1* | Beclin 1, autophagy related | Rn00586976_m1 |
| *Ccl2* | Chemokine (C-C motif) ligand 2 | Mm00441242_m1 |
| *Col1a1* | Collagen, type I, alpha 1 | Mm00801666_g1 |
| *Col1a1* | Collagen, type I, alpha 1 | Rn01463848_m1 |
| *Col3a1* | Collagen, type III, alpha 1 | Mm01254476_m1 |
| *Col3a1* | Collagen, type III, alpha 1 | Rn01437681_m1 |
| *Gapdh* | Glyceraldehyde-3-phosphate dehydrogenase | Rn01775763_g1 |
| *Il6* | Interleukin 6 | Mm00446190_m1 |
| *Il6* | Interleukin 6 | Rn99999011_m1 |
| *Nppa* | Natriuretic peptide type A | Mm01255747_g1 |
| *Nppa* | Natriuretic peptide type A | Rn00664637_g1 |
| *Nppb* | Natriuretic peptide type B | Mm01255770_g1 |
| *Nppb* | Natriuretic peptide type B | Rn00580641_m1 |
| *Tgfb1* | Transforming growth factor, beta 1 | Mm01178820_m1 |
| *Tgfb1* | Transforming growth factor, beta 1 | Rn00572010_m1 |
| *Tnf* | Tumor necrosis factor | Mm00443258_m1 |
| *Tnf* | Tumor necrosis factor | Rn99999017_m1 |
| *Rn18s* | 18S ribosomal RNA | Mm03928990_g1 |
| *Sqstm1* | Sequestosome 1 | Mm00448091_m1 |
| *Sqstm1* | Sequestosome 1 | Rn00709977_m1 |
