# Supplementary material for: Pirfenidone restores metabolic hormones and cardiac autophagy via p-AMPK in MASH
Source: J Transl Med. 2026 Aug 19;24:1077. doi: 10.1186/s12967-026-08828-1 (PMC13495469; doi:10.1186/s12967-026-08828-1)
Supplement: Supplementary file 2 [file 12967_2026_8828_MOESM7_ESM.docx]

**Article title: Pirfenidone restores metabolic hormones and cardiac autophagy via p-AMPK in MASH**

**Journal:** **Journal of Translational Medicine**

**Authors:** Daniel López-Cifuentes ^1,2^, Ana Sandoval-Rodríguez ^1^, Rebeca Escutia-Gutiérrez ^1^, Juan Armendariz-Borunda^1,3^, Jorge Gutiérrez-Cuevas^1*^

^1^University of Guadalajara, Institute for Molecular Biology in Medicine and Gene Therapy, Department of Molecular Biology and Genomics, CUCS, Jalisco, México.

^2^Doctorate in Sciences in Molecular Biology in Medicine, University Center of Health Sciences, University of Guadalajara, Guadalajara 44340, Jalisco, Mexico.

^3^Tecnologico de Monterrey, Campus Guadalajara, Jalisco, México.

*Corresponding Author

Dr. Jorge Gutiérrez Cuevas

University of Guadalajara, Institute for Molecular Biology in Medicine and Gene Therapy, Department of Molecular Biology and Genomics, CUCS, Guadalajara, Jalisco, México.

Tel: +52-331-062-2083

E-mail: gutierrezcj05@gmail.com

**Supplementary Table S2** List of antibodies used to determine the relative protein levels via western blotting

| **Antibody name** | **Catalog** | **Company** |
| --- | --- | --- |
| BECN1 | ab62557 | Abcam |
| GAPDH | ab8245 | Abcam |
| LC3B | ab192890 | Abcam |
| p-AMPKα (Thr172) | 2535S | Cell Signaling |
| AMPKα | ab80039 | Abcam |
| SQSTM1 | ab109012 | Abcam |
